# Transcriptome reprogramming and myeloid skewing in haematopoietic stem and progenitor cells in systemic lupus erythematosus

**DOI:** 10.1136/annrheumdis-2019-215782

**Published:** 2019-11-28

**Authors:** Maria Grigoriou, Aggelos Banos, Anastasia Filia, Pavlos Pavlidis, Stavroula Giannouli, Vassiliki Karali, Dionysis Nikolopoulos, Antigone Pieta, George Bertsias, Panayotis Verginis, Ioannis Mitroulis, Dimitrios T Boumpas

**Affiliations:** 1 4th Department of Internal Medicine, Attikon University Hospital and Joint Rheumatology Program, National and Kapodestrian University of Athens, Athens, Greece; 2 Laboratory of Inflammation and Autoimmunity, Biomedical Research Foundation, Academy of Athens, Athens, Greece; 3 Institute of Computer Science, Foundation of Research and Technology Hellas, Heraklion, Greece; 4 2nd Department of Internal Medicine, Hippokrateion Hospital, National and Kapodestrian University of Athens, Athens, Greece; 5 Department of Rheumatology, Clinical Immunology and Allergy, School of Medicine, University of Crete, Heraklion, Greece; 6 Laboratory of Immune Regulation and Tolerance, Biomedical Research Foundation of the Academy of Athens, Athens, Greece; 7 Department of Hematology and Laboratory of Molecular Hematology, Department of Medicine, Democritus University of Thrace, Alexandroupolis, Greece; 8 Institute for Clinical Chemistry and Laboratory Medicine, Center of Internal Medicine, University Hospital of Dresden, Dresden, Germany; 9 Rheumatology-Clinical Immunology Unit, Medical School, University of Cyprus, Nicosia, Cyprus

**Keywords:** systemic lupus erythematosus, autoimmunity, inflammation

## Abstract

**Objectives:**

Haematopoietic stem and progenitor cells (HSPCs) are multipotent cells giving rise to both myeloid and lymphoid cell lineages. We reasoned that the aberrancies of immune cells in systemic lupus erythematosus (SLE) could be traced back to HSPCs.

**Methods:**

A global gene expression map of bone marrow (BM)-derived HSPCs was completed by RNA sequencing followed by pathway and enrichment analysis. The cell cycle status and apoptosis status of HSPCs were assessed by flow cytometry, while DNA damage was assessed via immunofluorescence.

**Results:**

Transcriptomic analysis of Lin^−^Sca-1^+^c-Kit^+^ haematopoietic progenitors from diseased lupus mice demonstrated a strong myeloid signature with expanded frequencies of common myeloid progenitors (CMPs)—but not of common lymphoid progenitors—reminiscent of a ‘trained immunity’ signature. CMP profiling revealed an intense transcriptome reprogramming with suppression of granulocytic regulators indicative of a differentiation arrest with downregulation trend of major regulators such as *Cebpe*, *Cebpd* and *Csf3r*, and disturbed myelopoiesis. Despite the differentiation arrest, frequencies of BM neutrophils were markedly increased in diseased mice, suggesting an alternative granulopoiesis pathway. In patients with SLE with severe disease, haematopoietic progenitor cells (CD34^+^) demonstrated enhanced proliferation, cell differentiation and transcriptional activation of cytokines and chemokines that drive differentiation towards myelopoiesis, thus mirroring the murine data.

**Conclusions:**

Aberrancies of immune cells in SLE can be traced back to the BM HSPCs. Priming of HSPCs and aberrant regulation of myelopoiesis may contribute to inflammation and risk of flare.

**Trial registration number:**

4948/19-07-2016.

**Key messages**

**What is already known about this subject?**

- Most cells participating in the pathogenesis of systemic lupus erythematosus (SLE) originate from bone marrow (BM) haematopoietic stem and progenitor cells (HSPCs). HSPCs actively respond to inflammatory stimuli by myeloid skewing, but this may lead to exhaustion, decreased function, increased risk for inflammation, decreased adaptive immunity and increased cardiovascular mortality.

**What does this study add?**

- In SLE, there is evidence of deregulation of haematopoiesis with skewing towards the myeloid lineage at the expense of lymphopoiesis and priming of HSPCs that exhibit a ‘trained immunity’ signature; this may contribute to inflammation and risk of flare.

**How might this impact on clinical practice or future developments?**

- Abnormalities of immune cells in SLE can be traced back in the BM HSPCs, a disease where stem cell therapy has been considered for refractory cases. Re-establishment of the appropriate myeloid versus lymphoid balance and alleviation of cell exhaustion may improve transplantability of HSPCs and may restore immune function. This could also decrease risk of infection and atherosclerosis and attenuate inflammation, decreasing the risk of flare.

## Introduction

In systemic lupus erythematosus (SLE), an interplay between environmental, genetic and epigenetic factors leads to perturbation of complex biological networks culminating into diverse clinical phenotypes. In this disease, interferon-alpha (IFN-α)-driven immunological alterations result in persistent immune responses against autologous nucleic acids, mimicking a sustained antiviral response. Intractable tissue damage caused by autoantibodies or immune-complex depositions affects several organs, leading to significant morbidity.^1 2^

Haematopoietic stem and progenitor cells (HSPCs) represent the most primitive multipotent population giving rise to all blood cell types.^3^ HSPCs reside in the bone marrow (BM) niche and remain in a quiescent state. Under stress or inflammatory conditions, they respond by proliferating and differentiating to replenish any progeny needed.^4–6^

A key observation in SLE is that most cells participating in its pathogenesis, such as lymphocytes, monocytes and neutrophils, originate from HSPCs. In a congenic strain of lupus mice, the function of hematopoietic stem cells is altered by both genetic and inflammatory factors with evidence of increased self-renewal and resistance to stress.^7^ In patients with SLE with cytopenias, BM exhibits necrosis, stromal alterations, hypocellularity, dyspoiesis and distortion of normal architecture with abnormal localisation of immature precursors aggregates.^8^ Gene expression studies from our group have demonstrated upregulation of genes involved in cell death and granulopoiesis, providing further evidence of the role of apoptosis and granulocytes in its pathogenesis.^9^

Within the BM niche, inflammatory cytokines and myeloid-specific growth factors, including interleukin (IL)-1 and granulocyte macrophage growth factor,^10^ drive the reprogramming of HSPCs towards myeloid lineage by epigenetic modifications and induction of lineage-specific transcription factors. These alterations increase their adaptation to inflammatory and haematopoietic stress, promoting the generation of myeloid cells that confer protection to secondary infection, in a phenomenon termed ‘trained innate immunity’ or ‘innate immune memory’.^11 12^ Perturbed immunological imprinting (mediated by hyperactive and myeloid priming) could be detrimental, exaggerating immune responses in autoimmune and inflammatory diseases such as arthritis,^13 14^ SLE^15^ or atherothrombosis.^16–18^

We reasoned that the fundamental molecular aberrations in SLE (genetic or epigenetic) may be traced back in the HSPCs within the BM. To this end, we used the NZBW/F1 mouse model of SLE to investigate the lupus transcriptome of HSPCs and compared it with transcriptomic data from human SLE CD34^+^ cells. Herein, we report reprogramming of HSPCs towards myeloid lineage—with evidence of a ‘trained immunity’ signature—and propose that this may contribute to exaggerated immune responses and flares in SLE.

## Methods

### Mice

C57BL/6 mice were purchased from the Jackson Laboratory. NZB/OlaHsd and NZW/OlaHsd mice were purchased from Envigo. NZBW/F1 mice were considered diseased, when exhibiting ≥100 ng/dL of urine protein after the completion of 6 months of their life.^19^ Age-matched female mice were used (B6-young (B6-Y)/F1-prediseased (F1-P): 12 and B6-old (B6-O)/F1-lupus (F1-L): 28–36 weeks old). All animals were maintained in the BRFAA animal facility.

### Flow cytometry and cell sorting

Single-cell suspensions were prepared from BM, peripheral blood mononuclear cells (PBMCs) or spleen and were stained with conjugated antibodies. CD11b^−^Gr-1^−^Ter119^−^B220^−^CD16/32^−^Sca-1^+^c-Kit^+^ (Lin^−^Sca-1^+^c-Kit^+^ (LSK)) and CD11b^−^Gr-1^−^Ter119^−^B220^−^CD16/32^−^CD34^+^Sca-1^+^c-Kit^+^ (common myeloid progenitor (CMP)) cells were isolated from BM (tibia, femur and brachial) and sorted on a FACS-ARIA-III (Becton Dickinson Biosciences). Cell purity was ≥95%. Data were analysed with FlowJo.

### Human subjects

BM aspirates and PBMCswere obtained from SLE and gender matched healthy controls (HCs). Patients met the 1999 American College of Rheumatology revised criteria for the classification of SLE.^20^ Patients’ clinical and serological characteristics are summarised in online supplementary tables 1 and 5.

10.1136/annrheumdis-2019-215782.supp1 Supplementary data

### Mononuclear cell isolation and processing

Bone marrow mononuclear cells (BMMCs) were isolated using Histopaque 1077 (Sigma-Aldrich). BMMCs were washed, and erythrocytes were lysed with red blood cell buffer (420301, Biolegend) and stained with conjugated antibodies. CD34^+^ cells were isolated from BMMCs using magnetic beads (18056, StemCell Technologies).

### RNA sequencing

LSKs and CMPs were sorted from BM of NZBW/F1 and C57BL/6 mice. CD34^+^ cells were isolated from BMMCs. Genes with a false discovery rate of ≤0.05 and a fold change of >1.5 were considered statistically significantly upregulated/downregulated, respectively. For the human–mouse comparison, human genes were converted to mouse orthologos and pathways were compared based on their ID.

### Statistics

Statistical analyses were performed using unpaired two-tailed Student’s t-test, while Mann-Whitney U test was used for the comparison of two groups. Data are presented as mean±SD. Differences were considered statistically significant at p<0.05. All data were analysed using GraphPad Prism V.5 software.

### Study approval

Informed consent was obtained from all patients and HC prior to sample collection (Athens, Greece, protocol 10/22-6-2017). All procedures in mice were in accordance with institutional guidelines and were reviewed and approved by the Greek Federal Veterinary Office (Athens, Greece).

## Results

### The transcriptional profile of murine lupus HSPCs demonstrates myeloid skewing

**Figure 1:**
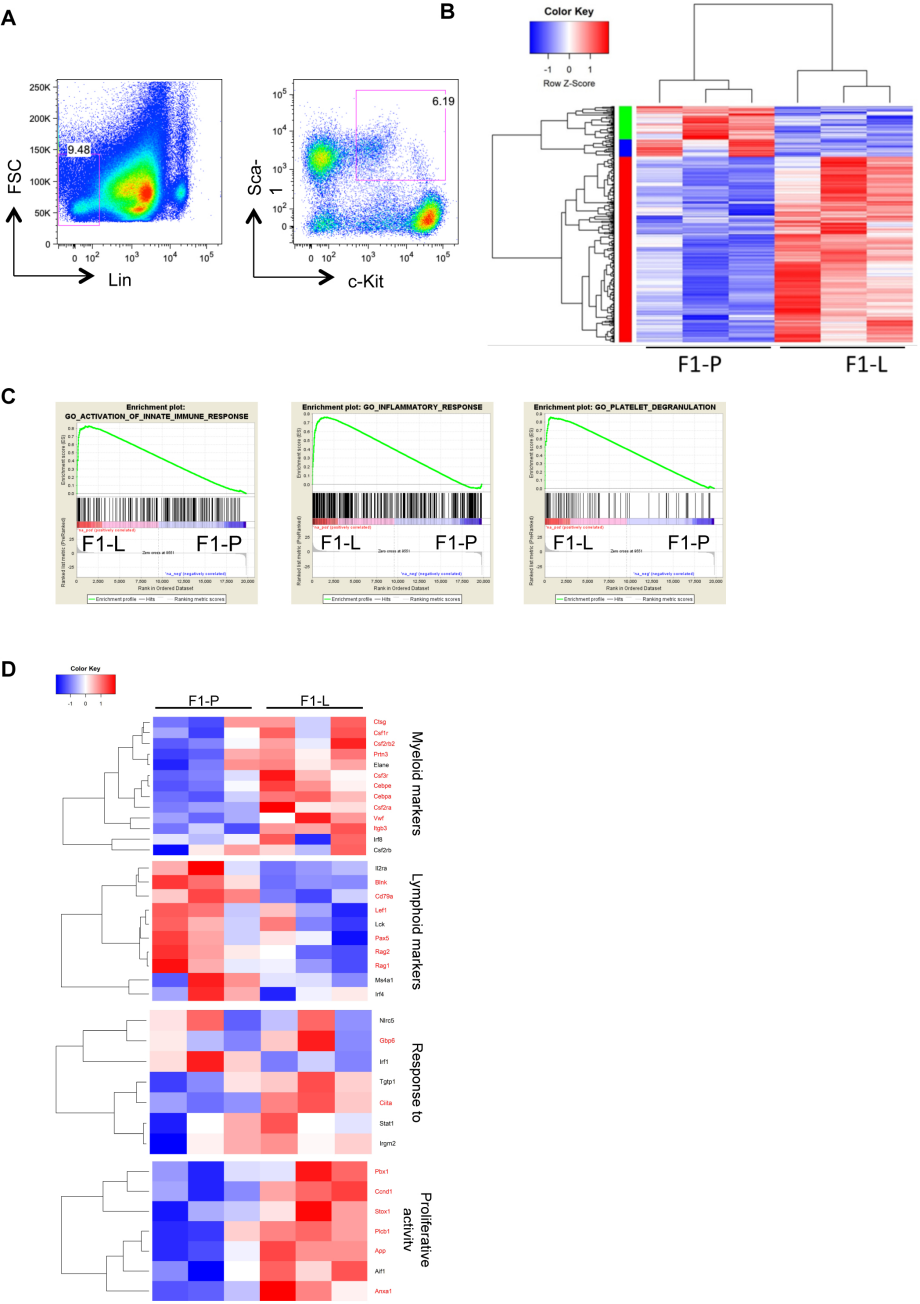
Transcriptional profiling of murine LSK by RNA sequencing demonstrates myeloid skewing. (A) Representative fluorescence-activated cell sorting plots for the identification of haematopoietic stem and progenitor cells. After gating for Lin^−^ cells, LSK were characterised as c-Kit^+^Sca-1^+^. (B) Heatmap of DEGs (|FC|>1.5, FDR<0.05) in BM-derived LSK between F1-P and F1-L mice (n=3 replicates per group). (C) GSEA plot showing the enrichment of ‘GO activation of innate immune response’ (NES 1.62, FDR 0.002), ‘GO inflammatory response’ (NES 1.57, FDR 0.022) and ‘GO platelet degranulation’ (NES 1.60, FDR 0.009) gene sets in LSK F1-L mice. (D) Heatmaps of genes related to myelopoiesis, lymphopoiesis, IFN response and enriched GSEA term ‘GO positive regulation of cell cycle phase transition’ (FDR 0.16) in BM-derived LSK F1-P and F1-L mice. Genes with p<0.05 are marked in red. BM, bone marrow; DEG, differentially expressed gene; FC, fold change; FDR, false discovery rate; FSC, forward scatter; F1-L, F1-lupus; F1-P, F1-prediseased; GSEA, gene set enrichment analysis; IFN, interferon; LSK, Lin^−^Sca-1^+^c-Kit^+^; NES, normalised enrichment score.

To study whether HSPCs in SLE exhibit transcriptional alterations, we used the spontaneous mouse model NZBW/F1^21–23^ at two time points: *preclinical* stage (F1-P) and *clinical stage*, defined as the point with proteinuria of >100 ng/dL (F1-L). Age-matched female C57BL/6 mice served as controls (B6-Y and B6-O, respectively). Gene profiling was performed in murine LSK compartment—representing HSPCs in mice—sorted by flow cytometry from BM of NZBW/F1 lupus and C57BL/6 control mice (figure 1A). A total of 758 differentially expressed genes (DEGs) between F1-P and F1-L mice were identified (figure 1B and online supplementary table 2), including enriched GO terms per cluster: haematopoietic cell lineage, neutrophil degranulation and cell adhesion (red); and lymphocyte activation, extracellular region and immunoglobulin heavy chain variable region genes (IGVH) repertoire (green/blue). Gene set enrichment analysis (GSEA) showed a positive correlation with signatures related to inflammatory response, activation of innate immune response and platelet degranulation (figure 1C and online supplementary table 3) in F1-L mice. These categories are crucial for the adaptation of stem cell phenotype to inflammation with studies showing expansion of stem cell-like megakaryocyte committed cells within HSPCs under inflammatory conditions.^24^ *Gene ontology and pathway analysis* demonstrated DEGs implicated in myeloid leukocyte-mediated immunity, cytokine secretion, granulocyte/neutrophil activation and migration in F1-L mice (online supplementary figure 1A). Notably, F1-L LSK demonstrated increased proliferation and strong myeloid signature (figure 1D). IFN-associated genes (*Gbp6* and *Ciita*) were upregulated in F1-L LSK, showing a strong type I IFN signature, a hallmark of active SLE^25^ (figure 1D). Comparison with publicly available gene sets—encompassing CMP^26^ and granulocyte–macrophage progenitor (GMP)^27^ signatures from wild-type mice as reference—demonstrated positive enrichment in F1-L LSK for CMP and GMP signatures (online supplementary figure 1B) indicative of priming towards this direction. Together, these transcriptomic data indicate increased proliferation and strong differentiation of F1-L LSK towards myeloid/granulocytic lineage.^28^

10.1136/annrheumdis-2019-215782.supp2 Supplementary data

10.1136/annrheumdis-2019-215782.supp3 Supplementary data

### Active proliferation and replicative stress of murine lupus HSPCs cells

**Figure 2:**
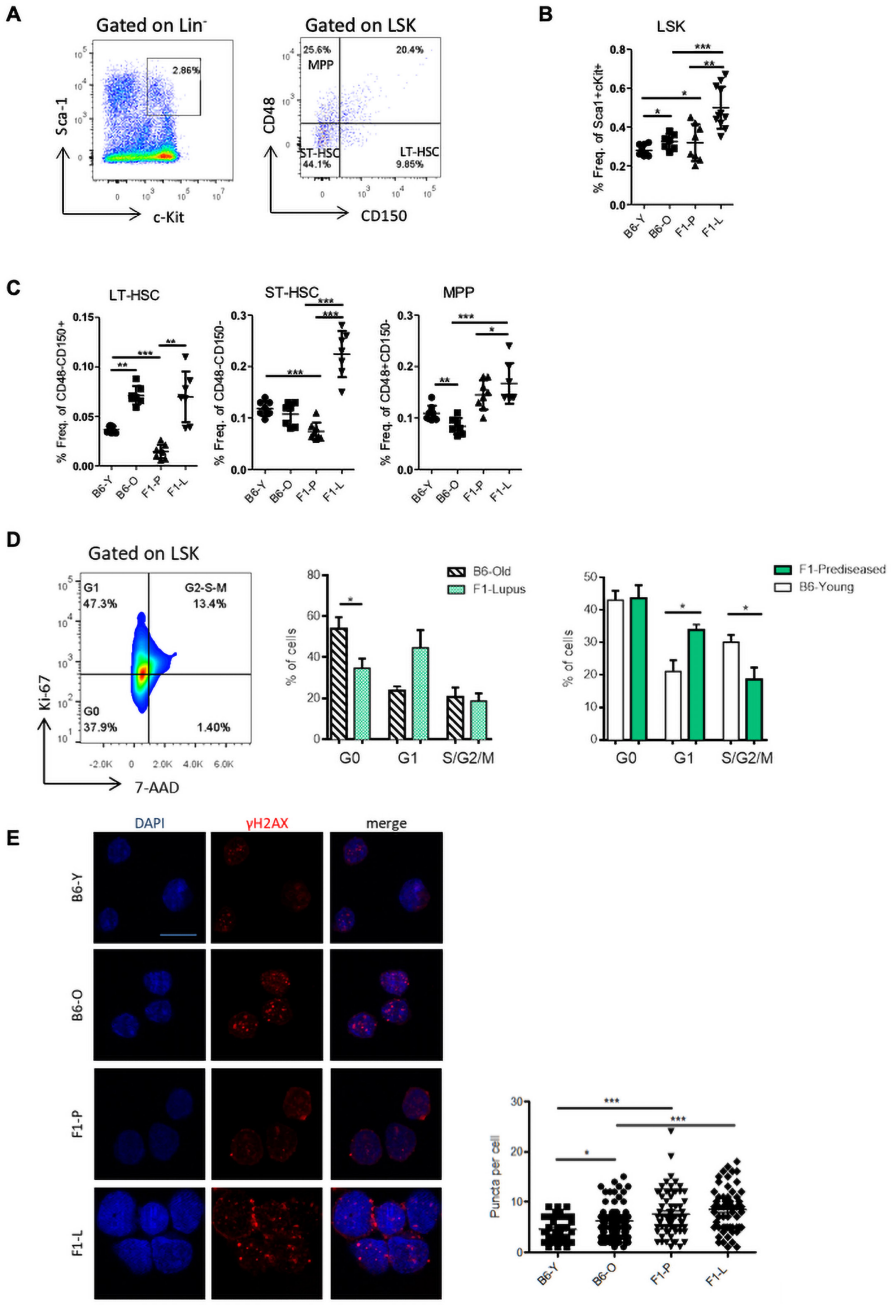
Phenotypical analysis of murine LSK by flow cytometry demonstrates enhanced proliferation. (A) Representative flow cytometry analysis within LSK compartment. (B) Frequencies of LSK in BM of F1-P, F1-L and their age-matched C57BL/6 control mice (n=7–10). (C) LT-HSCs, ST-HSCs and MPP in BM of F1-P, F1-L and their age-matched C57BL/6 control mice (n=7–10). (D) Representative flow cytometry plot of BM-derived LSK cell cycle analysis using Ki-67/7-AAD marker and frequencies of cells in each different phase of cell cycle (G0, G1 and S/G2/M) (n=4–6). (E) Representative confocal microscopy images for γ-H2AX (red) and DAPI (blue), and γ-H2AX puncta/cell in sorted LSK from BM of F1-P, F1-L and their age-matched C57BL/6 control mice (n=3–4, Leica TCS SP5 63x, scale bar: 10 µM). One representative experiment of four is shown. Results are mean±SEM. Statistical significance was obtained by unpaired Student’s t-test (*p≤0.05, **p≤0.01, ***p≤0.001). 7-AAD, 7-Aminoactinomycin D; BM, bone marrow; DAPI, 4′,6-diamidino-2-phenylindole; F1-L, F1-lupus; F1-P, F1-prediseased; LSK, Lin^−^Sca-1^+^c-Kit^+^; LT-HSC, Long Term-Hematopoietic Stem Cell; MPP, multipotent progenitor cell; ST-HSC, short term hematopoietic stem cell.

To validate transcriptomic data, we analysed the BM LSK compartment and its subpopulations. LSK were increased by almost twofold in F1-L mice compared with F1-P (figure 2A, B). Within the LSK compartment, short-term HSCs and multipotent progenitor cells (MPPs) had higher frequency in F1-L mice (figure 2A, C). Compared with their control counterparts, we found enhanced frequency of circulating LSK in the peripheral blood—but not in the spleen—of F1-L mice (online supplementary figure 1C, D), suggesting that BM-derived LSK may be activated, may exit the niche and then migrate to the periphery.

During steady state, HSPCs are relatively quiescent, maintaining a low number of cycling cells that will differentiate into mature blood cells.^29^ Cell cycle analysis of lupus LSK revealed increased proliferation with fewer F1-L LSK in G0 phase compared with B6-O control (figure 2D). The frequency of apoptotic LSK from NZBW/F1 mice was also increased compared with B6 with no significant difference in F1-P versus F1-L stage (online supplementary figure 1E). In view of the enhanced proliferation, γ-H2AX was measured as a marker of proliferative stress. Lupus LSK exhibited increased double-strand DNA breaks compared with B6 (figure 2E), suggesting that LSK in lupus mice is under replicative stress (see discussion).

### Differentiation arrest of CMPs with lupus disease progression

**Figure 3:**
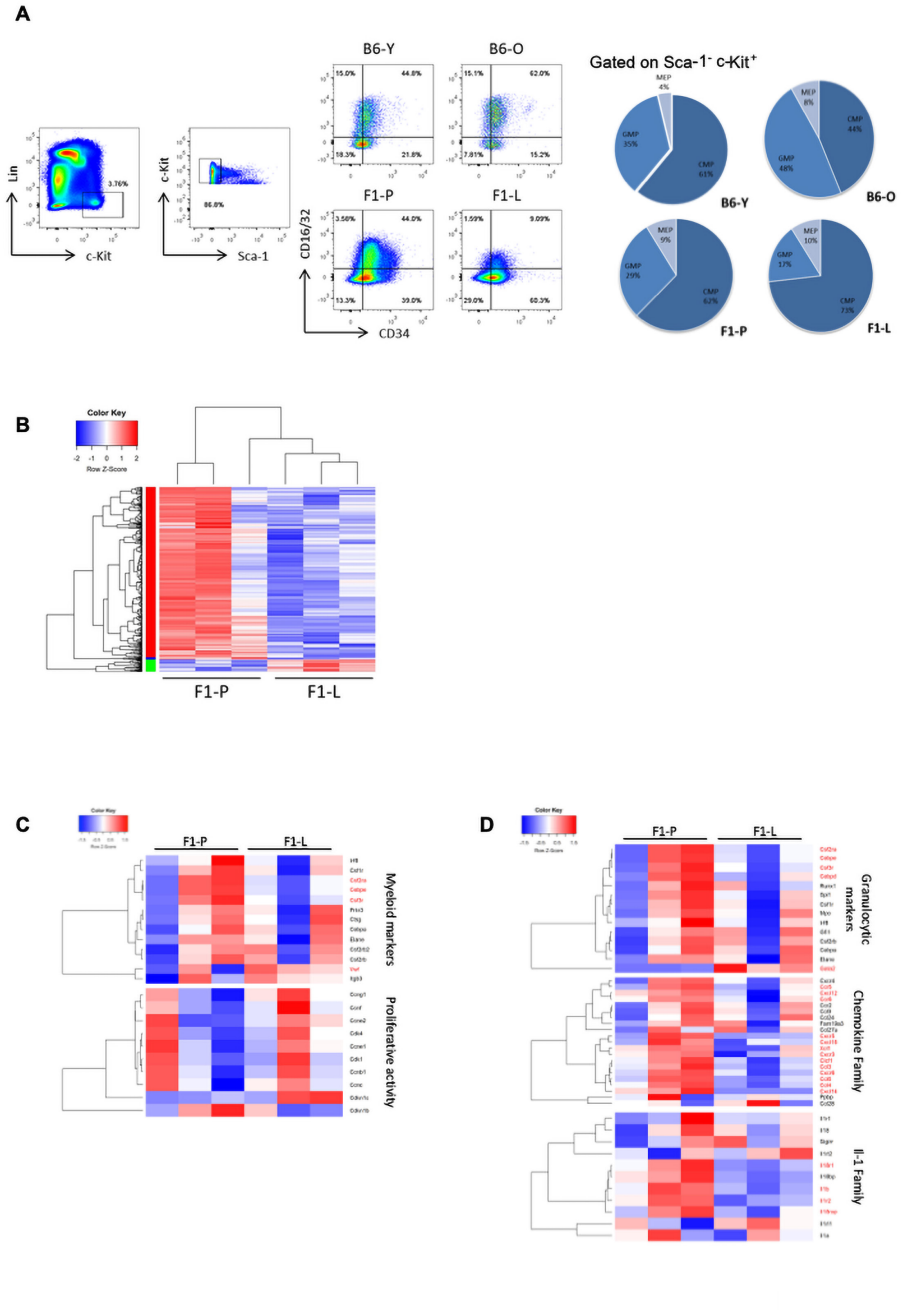
Attenuation of murine lupus CMP differentiation with the progression of the disease. (A) Representative flow cytometry analysis and frequencies of BM-derived CMPs (CD34^+^CD16/32^−^) and GMPs (CD34^+^CD16/32^+^) of F1-P, F1-L and their age-matched C57BL/6 control mice (n=8–10). (B) Heatmap of DEGs (|FC|>1.5, FDR<0.05) in BM-derived CMP cells between F1-P and F1-L mice (n=4 per replicate). (C) Heatmaps of genes related to myelopoiesis and proliferation in BM-derived CMP F1-P and F1-L mice. Genes with p<0.05 are marked in red. (D) Heatmaps of genes related to granulopoiesis, chemokine-related and IL-1-related factors in BM-derived CMP of F1-P and F1-L mice. Genes with p<0.05 are marked in red. BM, bone marrow; CMP, committed myeloid progenitor; FC, fold change; FDR, false discovery rate; F1-L, F1-lupus; F1-P, F1-prediseased; GMP, granulocyte–macrophage progenitor; IL, interleukin.

To delineate the haematopoietic differentiation after the LSK stage, we characterised the progenitors of each lineage. CMPs were increased by 2.5-fold (**p≤0.01), while GMPs were 2-fold (***p≤0.001) reduced in F1-L versus F1-P mice (figure 3A). There was no significant difference in common lymphoid progenitors (online supplementary figure 2A). Both LSK and CMP frequencies were increased in F1-L mice, a profile reminiscent of emergency granulopoiesis.^30^

To further investigate the regulation of myeloid differentiation, we performed transcriptional analysis. Most of 721 DEGs were downregulated at F1-L CMPs (figure 3B and online supplementary table 2), including enriched GO terms per cluster: response to IFN-beta and nucleotide signalling (green); immune response/immunoglobulins (blue); and cytokine signalling, neutrophil degranulation and haematopoietic cell lineage (red). Myeloid markers were downregulated and proliferation markers were not differentially expressed (figure 3C). DEGs were involved in pathways related to myeloid-mediated immunity, granulocyte activation, neutrophil migration and complement activation (online supplementary figure 2B). Thus, we checked the expression of specific granulocytic markers (figure 3D). Chemokines and regulators of IL-1 family^11 31 32^ were downregulated in F1-L CMPs. Indicatively, the expression of main regulators such as *Cebpe*, *Cebpd*, *Csf3r* and *Csf2rα* was dampened in CMPs of F1-L stage (figure 3D). Collectively, these results suggest differentiation arrest at the level of myeloid progenitors.

### Increased neutrophils in the lupus BM: evidence of ‘granulocytic priming’

**Figure 4:**
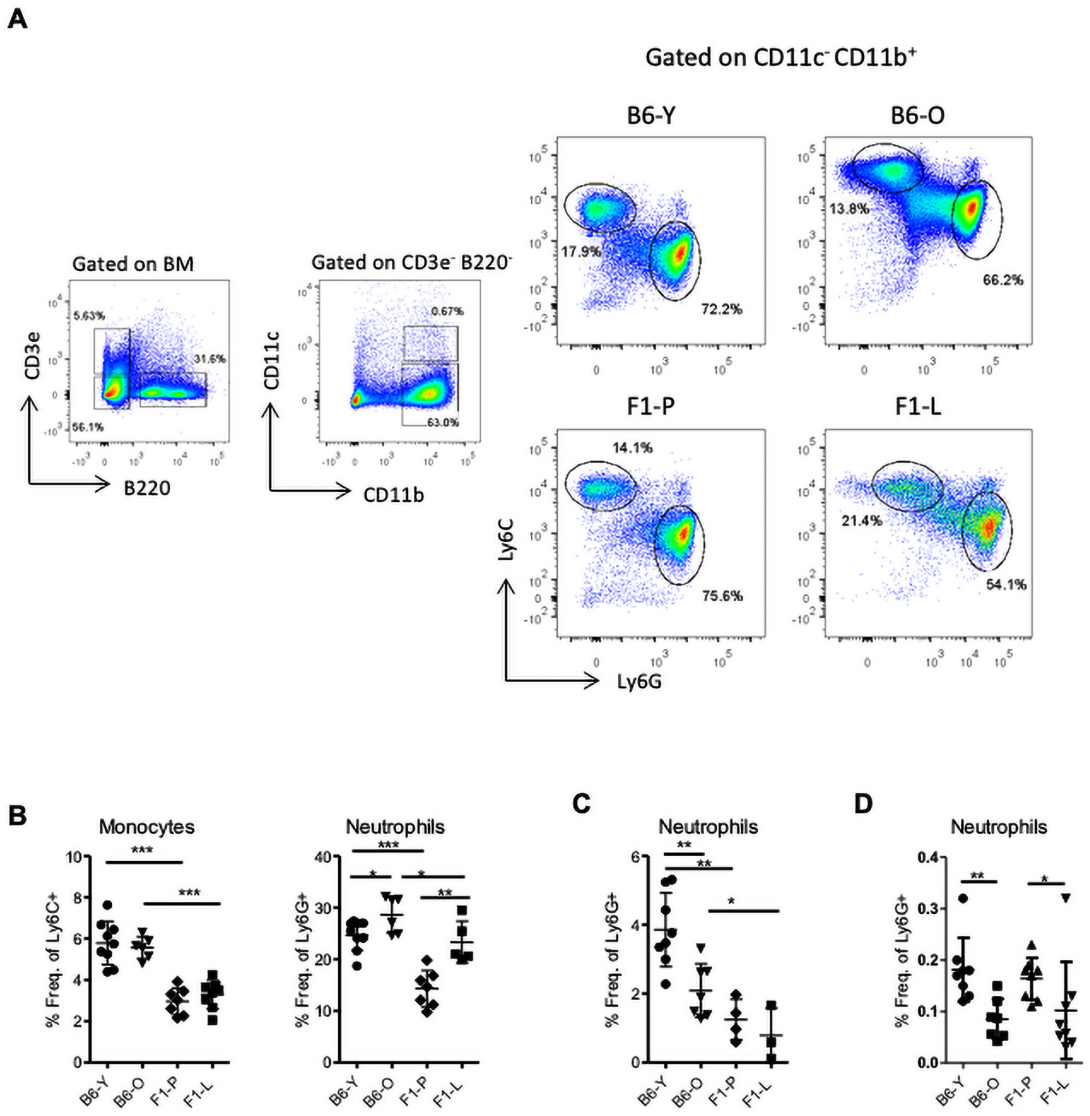
Neutrophils increase in the BM but decrease in the periphery of lupus mice. (A) Representative flow cytometry analysis of monocytes (CD3e^−^ B220^−^ CD11b^+^ Ly6C^+^) and neutrophils (CD3e^−^ B220^−^ CD11b^+^ Ly6G^+^) in BM of F1-P, F1-L and their age-matched C57BL/6 control mice. (B) Frequencies of monocytes and neutrophils in BM of F1-P, F1-L mice and their age-matched C57BL/6 control mice (n=6–11). (C) Frequencies of neutrophils in peripheral blood (n=3–8) and (D) spleen of F1-P, F1-L and their age-matched C57BL/6 control mice (n=6–10; *p≤0.05, **p≤0.01, ***p≤0.001). BM, bone marrow; F1-L, F1-lupus; F1-P, F1-prediseased.

In view of the differentiation arrest, we assumed that terminally differentiated cells may be decreased. However, neutrophils exhibited a 1.6-fold increase in the F1-L mice compared with F1-P, while there were comparable monocyte levels in BM (figure 4A, B). Ageing accounted for only an increase of 1.16-fold in neutrophils of control mice. In contrast, there was marked decrease of neutrophils in blood and spleen of F1-L mice (figure 4C, D, respectively), while monocytes did not differ significantly in the periphery (online supplementary figure 3A, B, respectively). Together these data suggest priming in the lupus BM towards neutrophils.

### Deregulation of differentiation of primed HSPCs indicates an alternative granulopoiesis pathway in lupus mice

To investigate how ‘granulocytic priming’ evolves during differentiation of haematopoiesis, we performed a comparative analysis between LSK and CMP transcriptomes. We used Regulatory Network Enrichment Analysis (RNEA) algorithm^33^ to report enrichment of transcription factors and regulators by combining previous studies with our data. We identified 13 common differentially expressed transcription factors and regulators (online supplementary figure 4A), predominantly downregulated in the F1-L CMP stage (online supplementary figure 4B), mainly of myeloid and granulocytic differentiation. Therefore, we looked into expression of major regulators of granulocytic and neutrophilic differentiation, such as *Cebpα*, *Cebpe*, *Irf8*, *Mpo* and *Elane*, and found them upregulated in F1-L LSK while downregulated in F1-L CMPs (online supplementary figure 4C). Collectively, these data indicate a deregulation of differentiation at the CMP level and ‘priming’ of LSK towards granulocytes, indicative of an *alternative granulopoiesis pathway*.

### Human SLE CD34^+^ transcriptome demonstrates active proliferation and myeloid skewing

**Figure 5:**
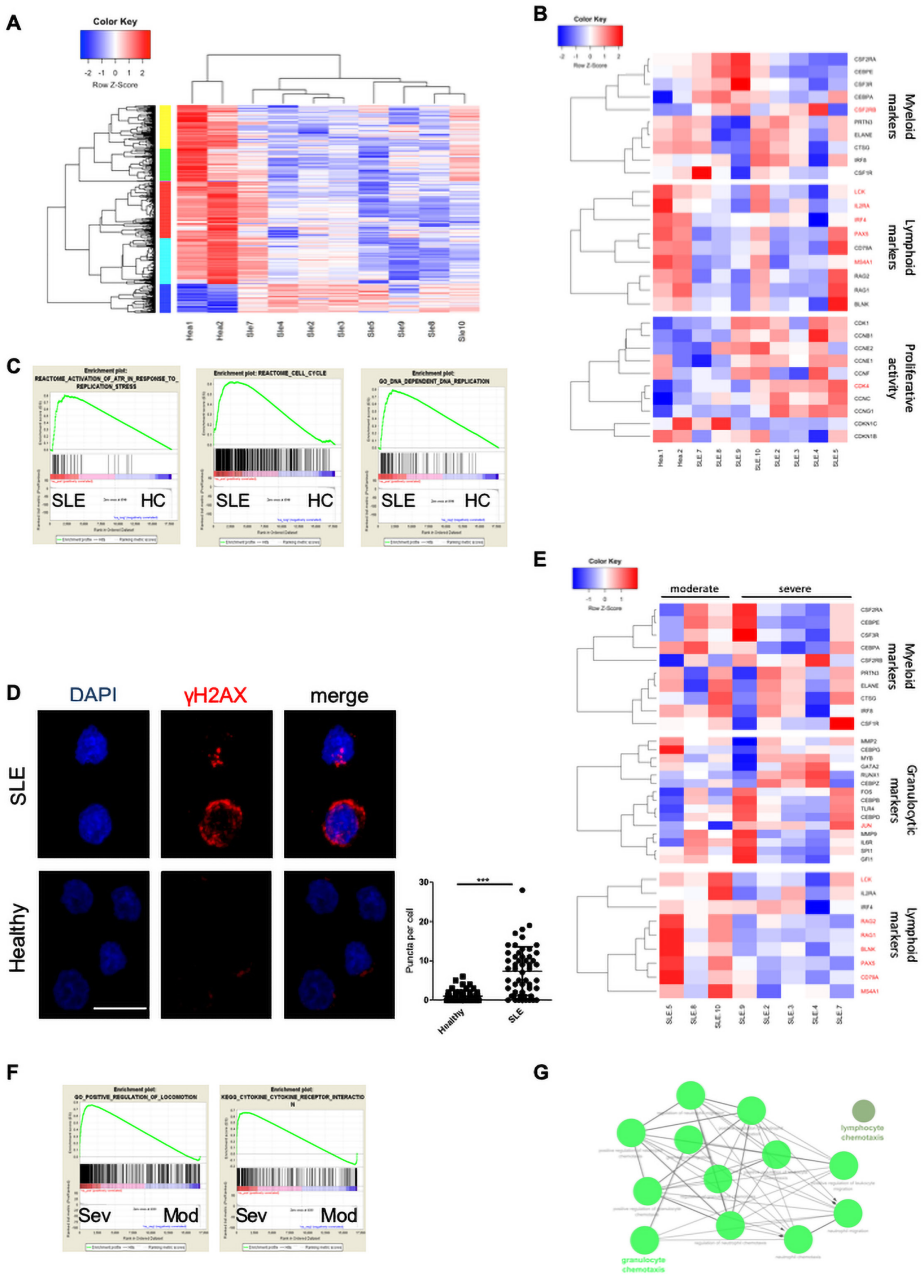
RNA sequencing of human CD34^+^ cells in patients with SLE suggests active proliferation with myeloid skewing. (A) Heatmap of DEGs in CD34^+^ cells isolated from BM of patients with SLE (n=8) and HC (n=2). (B) Heatmaps of genes related to myelopoiesis, lymphopoiesis and proliferation in patients with SLE and HC. Genes with p<0.05 are marked in red. (C) GSEA plot showing the enrichment of ‘reactome: activation of ATR in response to replication stress’ (NES 1.73, FDR 0.056), ‘reactome: cell cycle’ (NES 1.81, FDR 0.002), ‘GO DNA dependent DNA replication’ (NES 2.03, FDR<0.001) gene sets in CD34^+^ patient with SLE samples. (D) Representative confocal microscopy images for γ-H2AX (red) and DAPI (blue), and γ-H2AX puncta/cell in CD34^+^ cells from BM of patients with SLE (n=3) and HC (n=2) (Leica TCS SP5 63x, Scale bar: 10 µM). One representative experiment is shown. Results are mean±SEM. Statistical significance was obtained by unpaired Student’s t-test (*p≤0.05, **p≤0.01, ***p≤0.001). (E) Heatmaps of genes associated with myeloid, lymphoid and granulocytic markers in CD34^+^ cells isolated from BM of SLE with Mod (n=3) and Sev (n=5) disease. Genes with p<0.05 are marked in red. (F) GSEA plot showing the enrichment of ‘GO Positive regulation of locomotion’ (NES 1.58, FDR 0.0053) and ‘KEGG cytokine-cytokine receptor interaction’ (NES 1.30, FDR 0.042) gene sets in CD34^+^ SLE Sev patients. (H) Network of the upregulated genes associated with migration and chemotaxis of granulocytes and neutrophils using ClueGo plug-in in Cytoscape. BM, bone marrow; DAPI, 4′,6-diamidino-2-phenylindole; DEG, differentially expressed gene; FDR, false discovery rate; GSEA, gene set enrichment analysis; HC, healthy control; Mod, moderate; Sev, severe; SLE, systemic lupus erythematosus.

We next asked whether we could trace the ‘lupus LSK signature’ in human disease. To this end, we purified CD34^+^ cells from BM of female patients with SLE 34 35 (online supplementary tables 1 and 5) and HC. In humans, the CD34^+^ compartment comprises a cluster of 0.5%–2.0% of BM, which encompasses both stem and progenitor cells of different lineages.^36^ We identified 2364 DEGs between patients with SLE and HC, which contained 832 upregulated and 1532 downregulated genes (online supplementary table 2). Enriched GO terms per cluster include extracellular vesicle-mediated signalling in recipient (blue); myeloid leucocyte migration and chemokine signalling pathway (turquoise); integrin-mediated signalling and regulation of cell–cell adhesion (red); antigen processing and presentation, autoimmunity and abnormal inflammatory response (green); and cell surface receptor signalling pathway (yellow) (figure 5A). Lymphoid markers were downregulated in SLE, while expression of myeloid markers exhibited considerable variation within the patients. Combining these two panels, we found that early haematopoiesis in humans is characterised by skewing towards myeloid lineage (figure 5B). SLE CD34^+^ cells exhibited enhanced proliferation (figure 5B), while GSEA indicates a positive correlation with ‘activation of ATR in response to replication stress’, ‘cell cycle’ and ‘DNA-dependent DNA replication’ sets (figure 5C and online supplementary table 4). In view of enhanced proliferation,^37^ γ-H2AX was assessed to check if CD34^+^ cells are in proliferative stress; indeed, SLE HSPCs exhibited increased double-strand DNA breaks (figure 5D). Collectively, CD34^+^ cells of patients with SLE exhibited enhanced proliferation with increased DNA damage and unbalanced differentiation towards myeloid axis.

10.1136/annrheumdis-2019-215782.supp4 Supplementary data

Subgroup analysis showed that patients with SLE with both severe and moderate disease (online supplementary figure 6) showed variable expression of myeloid markers (figure 5E). Notably, expression of specific markers for granulopoiesis, such as *CEBPZ*, *CEBPD*, *GATA2*, was increased in a subgroup of patients with severe SLE compared with those with moderate disease (figure 5E), a result consistent with our findings in murine lupus LSK. GSEA revealed a positive correlation with ‘cytokine–cytokine receptor interaction’ and ‘positive regulation of locomotion’ sets in patients with severe SLE (figure 5F and online supplementary table 4). Enrichment analysis using upregulated genes in severe SLE revealed an over-representation of chemotaxis and migration of granulocytes and neutrophilic GO terms (figure 5G). Together, HSPCs are activated and more proliferative in patients with SLE compared with HC with a distinct transcriptional differentiation profile in patients with severe disease.

### Comparison of human lupus CD34^+^ with murine lupus CMP transcriptome reveals common attributes with evidence of arrest at the progenitor stage

**Figure 6:**
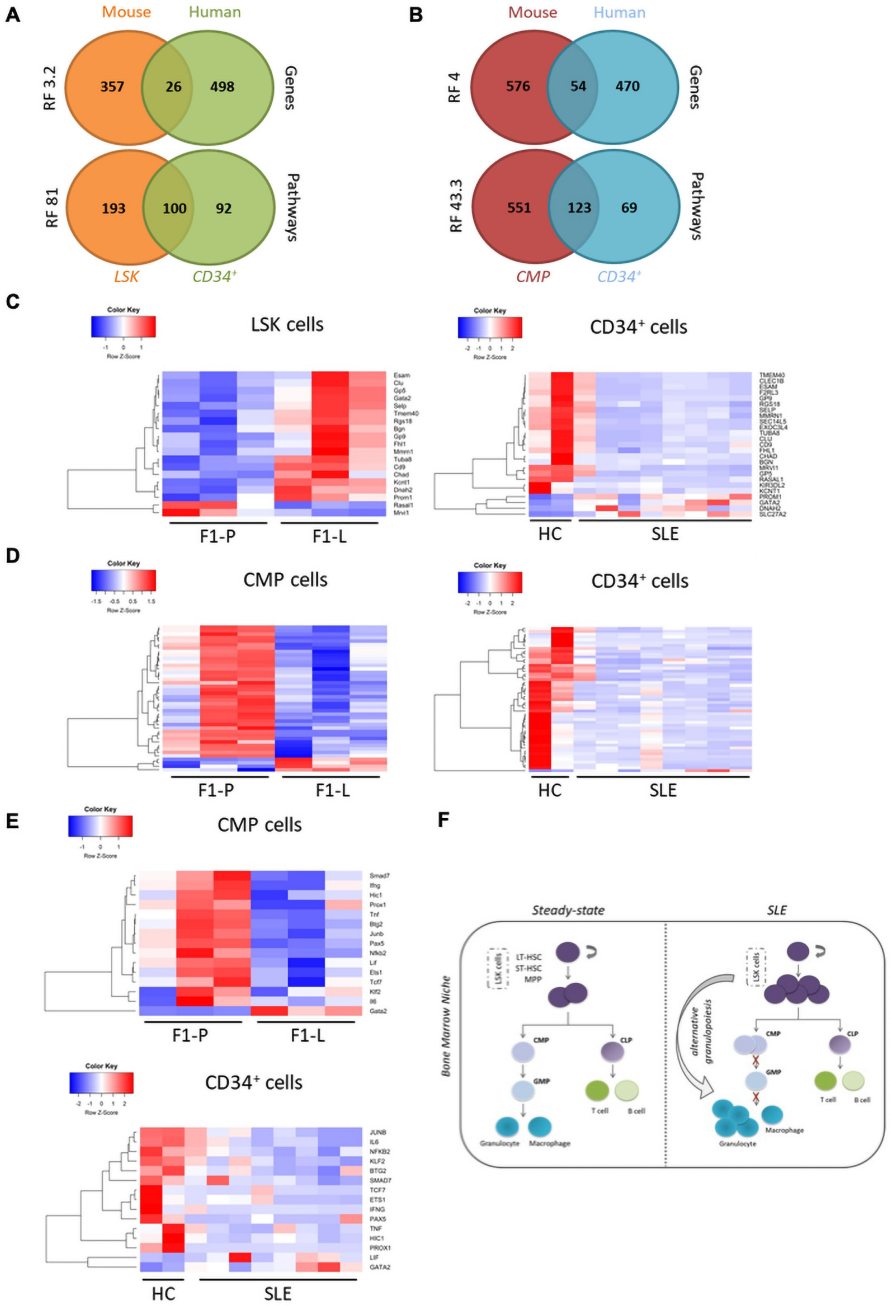
Comparison at the transcriptome level of human lupus CD34^+^ with murine lupus LSK and CMP reveals common attributes with evidence of arrest at the progenitor stage. (A) Venn diagrams showing the overlap between significant DEGs and the respective enriched GO terms and pathways in human CD34^+^ and mouse LSK. (B) Venn diagrams showing the overlap between significantly DEGs and the respective enriched GO terms and pathways in human CD34^+^ and mouse CMP cells. (C) Heatmaps of 26 common DEGs in murine LSK (left panel) and human CD34^+^ cells (right panel). (D) Heatmaps of 54 common DEGs in murine CMP cells (left panel) and human CD34^+^ cells (right panel). (E) Heatmaps of the enriched transcription factors and regulators in murine CMP cells (left panel) and CD34^+^ cells based on RNEA algorithm (right panel). (F) Proposed model of alternative granulopoiesis in SLE. LSK in SLE are predefined to differentiate towards the granulocytic lineage by skipping the CMP and GMP stages of the haematopoietic lineage. CLP, common lymphoid progenitor; CMP, committed myeloid progenitor; DEG, differentially expressed gene; HC, healthy control; LSK, Lin^−^Sca-1^+^c-Kit^+^; LT-HSC, long erm-hematopoietic stem cell; tSLE, systemic lupus erythematosus; MPP, multipotent progenitor cell; RNEA, Regulatory Network Enrichment AnalysisST-HSC, short term-hematopoietic stem cell.

Next, we compared human CD34^+^ transcriptome to LSK and CMP data.^38^ We found a significant overlap of LSK DEGs (6.7%) and CMPs DEGs (8.5%) with the human CD34^+^ DEGs (representation factor (RF)=3.2, p<2.7×10^−29^ and RF=4, p<4.7×10^−18^, respectively) (figure 6A, B). The majority of shared DEGs (26) between mouse LSK and human CD34^+^ cells were downregulated in patients with human SLE, but not in murine lupus (figure 6C). However, the expression of common DEGs (54) between mouse CMPs and human CD34^+^ cells exhibited commonalities (figure 6D). Thus, the SLE CD34^+^ transcriptomic profile is more reminiscent of murine lupus CMPs than murine lupus LSK. This is likely due to the fact that the haematopoietic stem cell signature might be diluted within the heterogeneous CD34^+^ population in humans.^39^

At pathway level, the overlap between mouse and human data was more prominent. Compared with gene-level results, there was a fivefold higher representation of the enriched pathways in LSK and a twofold higher representation of the enriched pathways in CMPs (RF=81, p<6.8×10^−172^, and RF=43.3, p<4.8×10^−178^, respectively), which were also found enriched in human HSPCs (figure 6A, B). Key pathways such as cell activation, regulation of cell differentiation, immune system development and leucocyte migration were shared. Using RNEA, we found 15 suppressed regulators in both lupus CMPs and CD34^+^ SLE cells. Among them, there were significant disease-specific effectors such as IFNγ and IL-6, as well as regulators for the homeostasis–differentiation balance of stem cells like Lif, Ets1 and Pax5 (figure 6E).

Collectively, human CD34^+^ cells from patients with SLE display more commonalities in their transcripthmic profile with murine lupus CMPs rather than LSK. This comparison indicates an arrest at the progenitor level both in murine and human lupus haematopoiesis as important regulators for terminal differentiation are downregulated.

## Discussion

Blood and immune cells derive from HSPCs, which reside in the BM in quiescent state, being ready to respond to stress, such as severe infection, systemic inflammation or iatrogenic myeloablation.^40^ Recent data suggest significant heterogeneity within the HSPCs with evidence of early lineage segregation, lineage-biassed existence and containment of lineage-restricted progenitors.^41^ These lineage-biassed and lineage-restricted cells within the phenotypical HSPC compartment might serve as an emergency backup for stress conditions, capable of efficiently and specifically counterbalance the sudden loss of a particular lineage. Herein, we provide evidence of dysregulated differentiation during haematopoiesis in SLE. Transcriptomic data demonstrate enhanced activation and differentiation preference towards myeloid/granulocytic lineage after disease onset. NZBW/F1 exhibit detectable levels of type I IFN compared with other SLE mouse models.^42^ We show that, indeed, IFN signature is present in the NZBW/F1 model and that lupus HSPCs can sense and respond to IFN. Chronic activation of the IFN-α pathway in HSPCs impairs their function, whereas acute IFN-α treatment promotes the proliferation of dormant HSPCs.^43^

Our flow cytometric analysis revealed enhanced proliferation of LSK in lupus mice and increase in their subpopulations. This finding is consistent with findings by Niu *et al* 7 in a congenic lupus model where they found a genetic polymorphism on the *Cdkn2c* gene related to cell cycle. In the context of stem cell proliferation and activation, Walter *et al* 37 showed direct response of HSPCs by exiting quiescence with concomitant DNA damage. In agreement to this, lupus LSK had more DNA damage compared with their controls, which could be detrimental for their maintenance and self-renewal. Pronounced cell cycle entry and consequent proliferative stress may result in impaired HSC self-renewal potential.^44 45^

To confirm the transcriptomic results on LSK differentiation, we profiled CMPs. Physiological myelopoiesis evolves through MPPs to lineage-restricted CMPs and then converges to GMPs.^46^ Phenotypical analysis of progenitors showed increased frequency of CMPs but decreased frequency of GMPs, as evidenced in RNA sequencing by ‘silencing’ of differentiation after the CMP stage. Myeloid skewing is, in part, expected due to inflammation and ageing,^47^ both operant in our model. Our results suggest ‘priming’ of LSK with a pronounced ‘myeloid/granulocytic signature’ but downregulation as the differentiation evolves towards canonical myelopoiesis (figure 6F).

Increased neutrophils in lupus BM suggest deregulation of homeostatic mechanisms in the level of CMPs with priming of LSK towards the granulocytic differentiation at the expense of lymphopoiesis. These results are consistent with our earlier findings of strong granulopoiesis signature in the BM by using DNA arrays.^9^ Priming of LSK highly correlates with the signature of HSPCs after ‘training’ with β-glucan^11^ (online supplementary figure 3), strongly indicating that ‘SLE inflammatory milieu’ promotes the immune training memory of BM progenitor cells. Accordingly, we found differentially methylated regions from lupus HSPCs overlapping with transcription factor binding sites relevant to haematopoietic development, including *Cebpα* (*data not shown*). Innate immune memory, while beneficial to host defence against pathogens, could also lead to maladaptation of the immune system in chronic inflammation, leading to perpetuation of chronic inflammatory disorders and predisposing to flares in response to environmental stimuli such as infections or stress.^48^

Myeloid cells are crucial for disease progression. In the periphery of lupus mice, we found increased circulating LSK but decreased neutrophils. This could be due to either extensive destruction of neutrophils in the periphery or migration to target tissues. This might act as a positive feedback loop where an inflammatory environment triggers priming and exit of HSPCs to periphery, driving them to increased myeloid output, which in turn circulates and perpetuates the inflammation as proposed by Oduro *et al* 14 in an arthritis mouse model. It is conceivable that neutrophils may migrate to the inflamed tissues, hence their relative paucity in the periphery. The release of neutrophil extracellular traps represents a novel neutrophil effector function contributing to thromboinflammation and fibrosis in SLE.^49^

It has been assumed that various blood cell lineages arise via a hierarchical scheme—starting with HSPCs—and that their differentiation potential becomes increasingly restricted through oligopotent and then unipotent progenitors. However, recent work suggests a developmental shift to an adult ‘two-tier’ hierarchy whereby HSPCs can generate restricted subsets of terminally differentiated progeny, bypassing the stepwise progression through common progenitor stage.^50^ Yammamoto *et al* 51 proposed a revised model of haematopoietic differentiation with the existence of progenitors within the HSPC compartment, mostly myeloid-committed ones. Our results are in agreement with this model. It is reasonable to assume that SLE LSK are already predefined to differentiate towards granulocytic lineage, creating an alternative granulopoiesis pathway in the haematopoietic tree (figure 6F). In parallel, differentiation arrest at the intermediate stage of CMPs blocks flow of haematopoiesis towards GMPs.

In summary, we have presented evidence for deregulation of granulopoiesis and priming of HSPCs, which may contribute to persistent inflammation in SLE and risk of flare once the disease is in remission. Myeloid skewing of HSPCs, associated with epigenetic tinkering, is also typical of HSPCs during ageing,^51 52^ contributing to decreased adaptive immunity and enhanced cardiovascular mortality of the elderly population.^53–55^ Re-establishment of the appropriate lymphoid versus myeloid balance in systemic autoimmune diseases may improve immune function, decreasing risk of infection or atherosclerosis and resolution of inflammation.
