# Supplementary material for: Transcriptome reprogramming and myeloid skewing in haematopoietic stem and progenitor cells in systemic lupus erythematosus
Source: Ann Rheum Dis. 2019 Nov 28;79(2):242–53. doi: 10.1136/annrheumdis-2019-215782 (PMC7025734; doi:10.1136/annrheumdis-2019-215782)
Supplement: Supplementary data [file annrheumdis-2019-215782supp001.pdf]

Supplemental material

Supplemental Figures

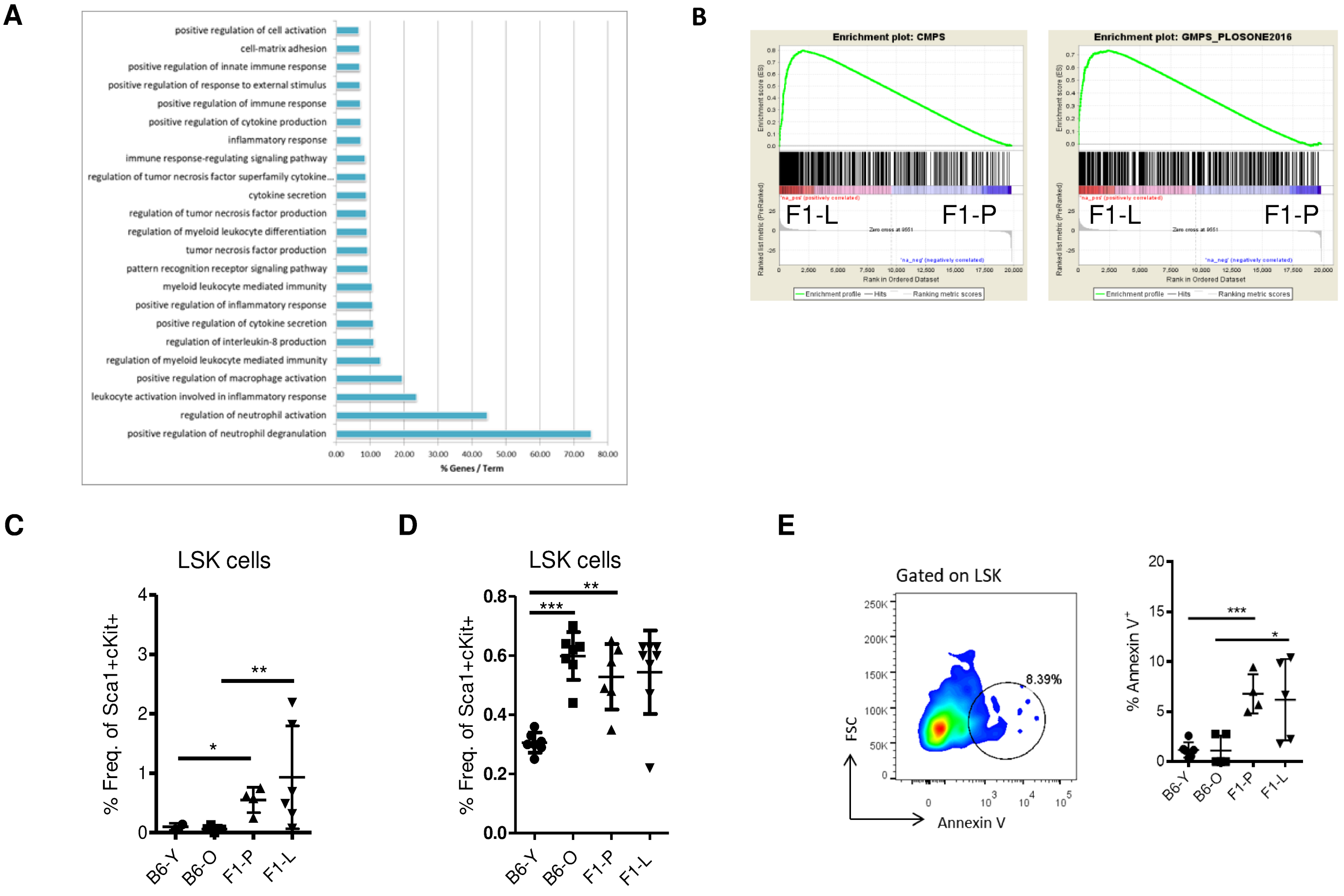

Supplemental Figure 1. Transcriptional and phenotypic profiling of murine LSK cells demonstrates activation of lupus LSK both in the bone marrow and periphery

(A) Gene ontology and pathway analysis of DEGs in BM-derived LSK cells between F1-P and F1-L mice using ClueGo plug-in in Cytoscape. (B) GSEA plot showing the enrichment of CMP signature (NES 1.66, FDR<0.001) and GMP signature (NES 1.52, FDR <0.001) genes in LSK F1-L transcriptome. (C) Frequencies of LSK in peripheral blood (*n*=2-6) and (D) spleen (*n*=6-10) of pre-diseased NZB/W F1, lupus NZB/W F1 and their age-matched C57BL/6 control mice. (E) Representative flow cytometry apoptosis analysis of BM-derived LSK cells and frequencies of Annexin V^{+} cells (*n*=4-6, *P≤0.05, **P≤0.01, ***P≤0.001).

Grigoriou M, *et al. Ann Rheum Dis* 2020; 79:242–253. doi: 10.1136/annrheumdis-2019-215782

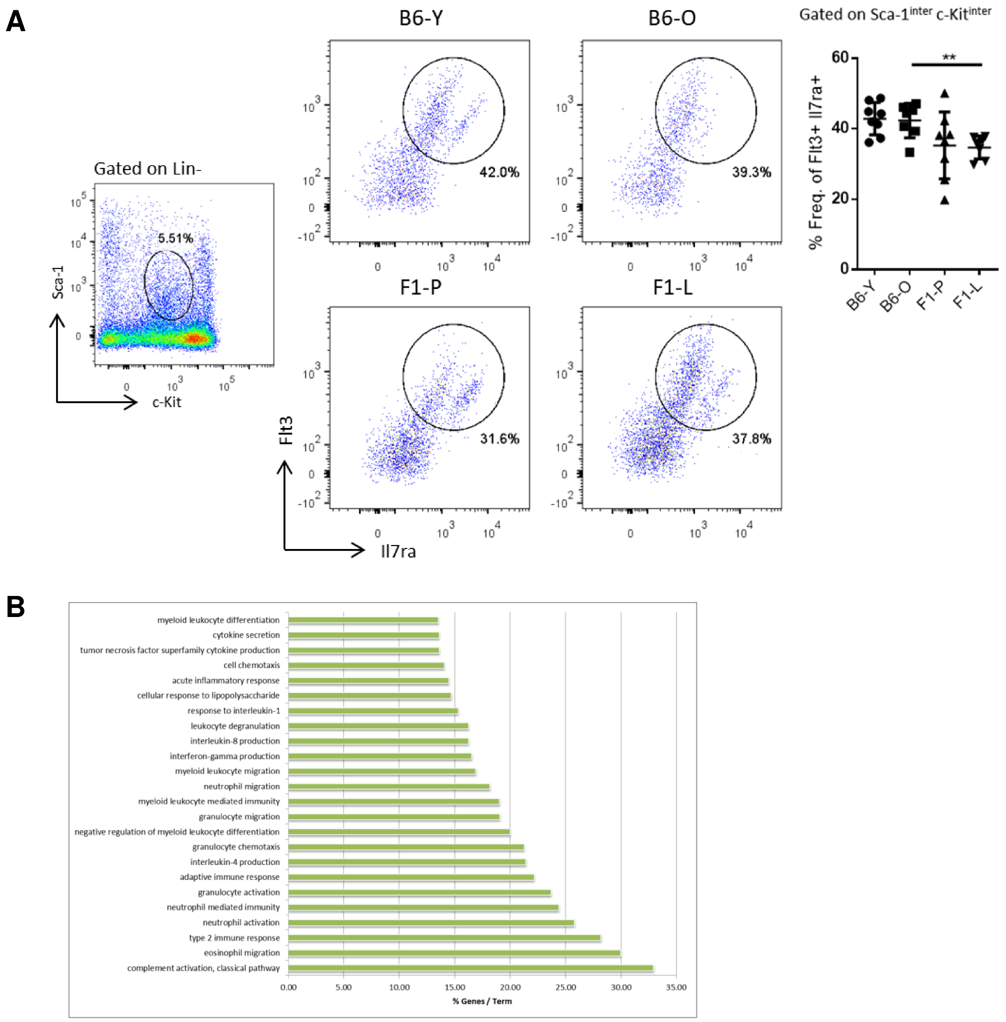

Supplemental Figure 2.

(A) Representative flow cytometry analysis and frequencies of BM-derived committed lymphoid progenitors (CLP) (Lin^- Sca-1^{inter} c-Kit^{inter} $Flt3^+$ Il7rα^+) of F1-P, F1-L and their age-matched C57BL/6 control mice (n=8-10, *P≤0.05, **P≤0.01, ***P≤0.001). (B) Gene ontology and pathway analysis of DEGs between BM-derived CMP cells F1-P and F1-L mice using ClueGo plug-in in Cytoscape.

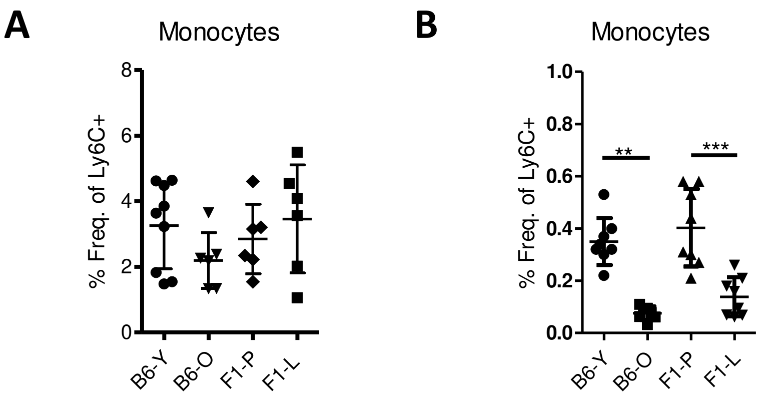

Supplemental Figure 3. Phenotyic analysis of monocytes in the bone marrow and in the periphery of lupus mice

(A) Frequencies of monocytes in peripheral blood and (B) spleen of pre-diseased NZB/W F1, lupus NZB/W F1 and their age-matched C57BL/6 control mice (n=6-10, *P≤0.05, **P≤0.01, ***P≤0.001).

Grigoriou M, *et al. Ann Rheum Dis* 2020; 79:242–253. doi: 10.1136/annrheumdis-2019-215782

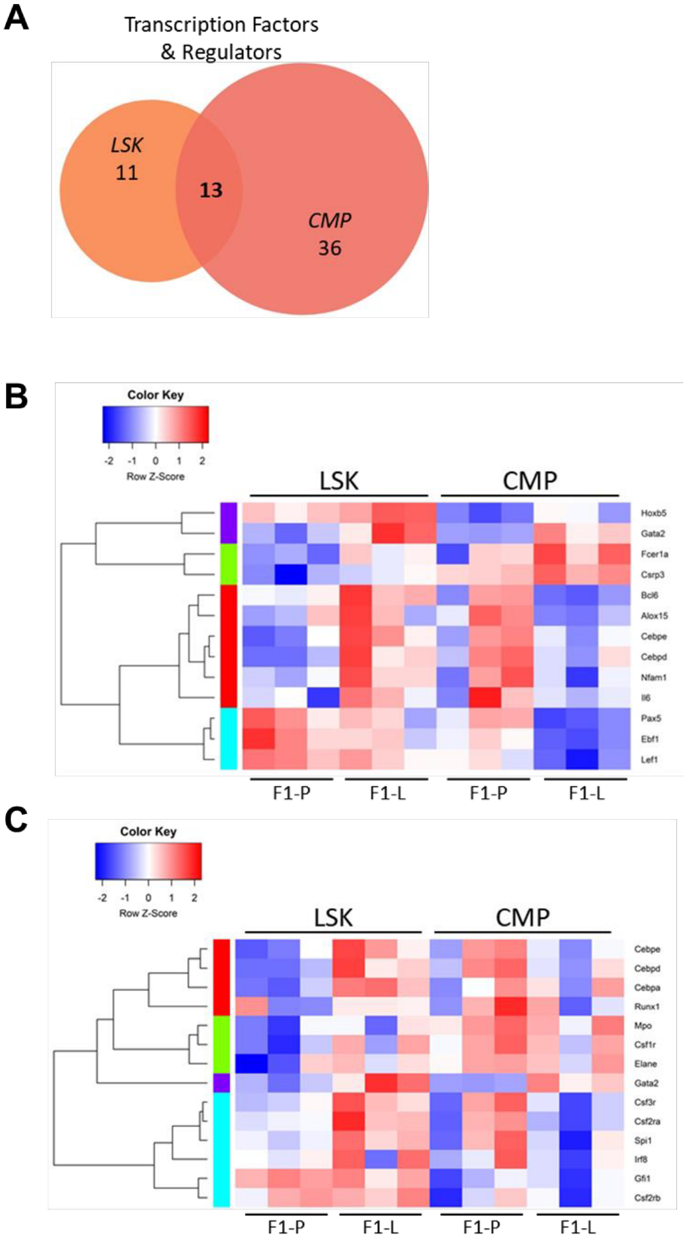

Supplemental Figure 4. Lupus LSK are programmed towards granulocytic differentiation.
(A) Venn diagram of the deregulated transcription factors and regulators of LSK and CMP in BM of F1-P and F1-L mice based on RNEA algorithm. (B) Heatmap of the common deregulated factors of LSK and CMP in BM of F1-P and F1-L mice. (C) Heatmap of differentially expressed granulocytic markers in BM-derived LSK and CMP cells of F1-P and F1-L mice.

Grigoriou M, *et al. Ann Rheum Dis* 2020; 79:242–253. doi: 10.1136/annrheumdis-2019-215782

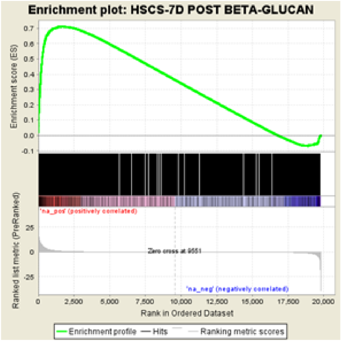

Supplemental Figure 5. Trained immunity-like phenotype of F1-L LSK in the bone marrow.
GSEA plot showing the enrichment of "HSCs 7 days post β-glucan' (NES 1.50, FDR<0.001) gene set in LSK F1-L mice. Trained immunity signature of HSCs was adopted by Mitroulis et al.

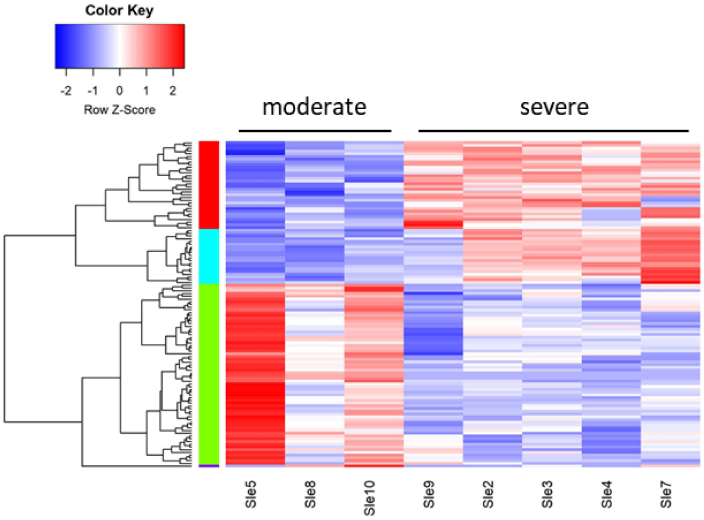

Supplemental Figure 6. Heatmap of DEGs in CD34^{+} cells isolated from BM of SLE with moderate (*n*=3) and severe (*n*=5) disease.

Grigoriou M, *et al. Ann Rheum Dis* 2020; 79:242–253. doi: 10.1136/annrheumdis-2019-215782

Supplemental Table 1

**Table 1. Clinical and demographic characteristics of SLE patients (*n=8*)**	
Sex, female/male	8/0
Age, mean ± SD	47.3 ± 16.02
SLEDAI*(mean ± SD)	8.12 ± 5.74
Severity pattern	
Moderate SLE	3/8
Severe SLE	5/8
History of Immunosuppressive Therapy	6/8
Nephritis	4 /8
NPSLE*	1/8
Serositis	3/8
Arthritis	7/8
Cytotoxic therapy	3/8
Corticosteroids	5/8
Hydroxychloroquine	7/8

*Footnote: SLEDAI, SLE disease activity index; NPSLE, neuropsychiatric SLE

Supplemental Table 2. Lists of the Differentially Expressed Genes
(1) DEGs between SLE patients and Healthy Controls in $CD34^+$ cells. (2) DEGs between SLE patients with severe and moderate disease in $CD34^+$ cells. (3) DEGs in LSK cells between F1-Lupus and F1-Prediseased NZB/W F1 mice. (4) DEGs in LSK cells between F1-Lupus and B6-Old mice. (5) DEGs from CMP cells from F1-Lupus and F1-Prediseased NZB/W F1 mice.

Supplemental Table 3. GSEA (using MSigDB Gene Set) on RNA-seq data from BM-derived LSK cells from NZB/W F1 pre-diseased and lupus mice.
NAME is the gene set name; SIZE is the number of genes in the gene set after filtering out those genes not in the expression dataset; ES is the enrichment score for the gene set; NES is the normalized enrichment score that accounts for size differences in gene sets; NOM p-val is the nominal p-value of ES significance based on permutation test; FDR q-val is the False Discovery Rate; FWER p-val is the family-wise error rate; RANK AT MAX is the position in the ranked list at which the maximum running enrichment score occurred.

Supplemental Table 4. GSEA (using MSigDB v6.1) on RNA-seq data from BM-derived $CD34^+$ cells from SLE patients and healthy controls, and patients with severe and moderate SLE.
NAME is the gene set name; SIZE is the number of genes in the gene set after filtering out those genes not in the expression dataset; ES is the enrichment score for the gene set; NES is the normalized enrichment score that accounts for size differences in gene sets; NOM p-val is the nominal p-value of ES significance based on permutation test; FDR q-val is the False Discovery Rate; FWER p-val is the family-wise error rate; RANK AT MAX is the position in the ranked list at which the maximum running enrichment score occurred.

Grigoriou M, *et al. Ann Rheum Dis* 2020; 79:242–253. doi: 10.1136/annrheumdis-2019-215782

Supplemental Table 5. Detailed clinical and serological items for each SLE patient.

Sample ID	Age	Proteinuria (mg)	Serum albumin levels (g/dl)	ANA titer	Anti-dsDNA & titer	C3/C4 levels	Nephritis	NPSLE	Serositis	Arthritis	SLEDAI	Severity pattern	Medication at Bone Marrow Aspiration	Cumulative dose of Glucocorticoids over the last month	Past Immunosuppressive medication
SLE.2	54	0	3	1:640	positive moderate	low	-	-	-	Yes	15	Severe	PRE (15mg), HCQ (400mg), CYC (6g)	315 mg	AZA, MMF, RTX
SLE.3	62	0	4.5	1:640	positive low	low	-	-	-	Yes	5	severe	PRE (20mg)	3,935 mg	RTX
SLE.4	82	2300	3.5	1:160	negative	low	+	-	+	Yes	14	Severe	PRE (15mg)	3,230 mg	None
SLE.5	35	0	3.9	1:1280	negative	low	-	-	-	Yes	1	Moderate	HCQ (200mg)	0 mg	None
SLE.7	50	0	3.9	1:1280	positive low	low	History of LN	History of NPSLE	History of serositis	Yes	4	Severe	PRE (10mg), MMF (1g)	300 mg	CYC, AZA
SLE.8	28	0	4	1:640	negative	low	-	-	-	Yes	4	Moderate	HCQ (400mg)	0 mg	CYC, AZA
SLE.9	42	8800	3.5	1:640	positive high	low	+		+	Yes	15	Severe	HCQ (400mg), Rituximab (1g), CYC (500mg),	5,100 mg	None
SLE.10	46	0	4.2	1:80	negative	low	-	-	-	Yes	8	Moderate	HCQ (400mg)	112 mg	None

*Footnote: AZA, Azathioprine; MMF, Mycophenolate Mofetil; RTX, Rituximab; CYC, Cyclophosphamide; PRE, Prednisone; HCQ, Hydroxychloroquine

Grigoriou M, *et al. Ann Rheum Dis* 2020; 79:242–253. doi: 10.1136/annrheumdis-2019-215782

Materials and methods

Flow cytometry

For murine analysis (catalog/clone): Ter119 (116206/TER-119), CD16/32 (101306/93, 101317/93), Gr1 (108406/RB6-8C5), B220 (103206/RA3-6B2), CD3e (100330,145-2C11), CD34 (119321/MEC14.7, 128608/HM34), Il7Rα (121114/SB/199), CD135 (135313/A2F10), CD150 (115909/TC15-12F12.2), CD48 (103426/HM48-1), Sca-1 (122512/E13-161.7, 108127/D7), c-Kit (105808/2B8), Ly6G (127608/1A8), Ly6C (128032/HK1.4), CD11c (117318/N418), CD11b (101212/M1/70), Ki-67 (652422/16A8, 652425/16A8), Annexin V/Annexin V Binding Buffer (640917/422201), 7-AAD Viability Staining Solution (420404) (Biolegend). For cell cycle intracellular staining, cells were fixed and stained using the Foxp3 Fixation & Permeabilization Kit (Molecular Probes) according to the manufacturer's instructions. *For human analysis* (catalog/clone): CD34 (343606/561), CD38 (356605/HB-7), CD45RA (HI100/304106), CD90 (328123/5E10), CD49f (313624/GoH3), CD10 (312217/HI10a), CD123 (306017/6H6), CD127 (351316/A019D5), CD4 (317428/OKT4), CD8 (344714/SK1), CD66b (305118/G10F5), CD14 (HCD14/325604), CD16 (3G8/302056), CD19 (HIB19/302241), CD25 (BC96/302604), HLA-DR (L243/307618). For neutrophils characterization, peripheral blood post erythrolysis was used.

Immunofluorescence

Cells were seeded in coverslips pretreated with poly-L-lysine (Sigma-Aldrich) for 15 minutes at 37°C and fixed with 4% paraformaldehyde (Sigma-Aldrich) for 15 minutes at room temperature. Cells were permeabilized by using 0.5% Triton-X 100 (Sigma-Aldrich), 2% BSA, stained with mouse anti-phospho-Histone H2A.X antibody (1:200; 05-636; Millipore), and incubated with Alexa Fluor 555 conjugated anti-mouse IgG (1:500; A28180; Invitrogen). DAPI staining (Sigma-Aldrich) was used for visualization of nuclei. Samples were coverslipped with mowiol and visualized using a ×63 oil lens in a Leica SP5 inverted confocal live cell imaging system. Numbers of γ-H2AX puncta/cell were calculated using a macro developed in Fiji software as previously described[1].

Human subjects selection

Exclusion criteria included: a) intake of morning glucocorticoid and/or immunosuppressive treatment; b) recent (within the last month) treatment with pulse intravenous methyl-prednisolone or cyclophosphamide; c) pregnancy; d) active infection or malignancy; e) concomitant auto-inflammatory or rheumatic disease. Severity of SLE was based on British Isles Lupus Assessment Group (BILAG) score combined with physician assessment at any time during the course of the disease (group A manifestations defined as severe disease, group B as moderate disease and group C-E as mild disease)[2].

RNA sequencing pipeline

Total RNA was extracted as described by manufacturer (NucleoSpin® RNA XS) and mRNA libraries were generated using the Illumina TruSeq Sample Preparation kit v2. Single-end 75-bp mRNA sequencing was performed on Illumina NextSeq 500. Quality of sequencing was assessed using FastQC software[3]. Raw reads in fastq format were collected and aligned to the mouse genome (mm10 version) and human genome (hg38 version) using STAR 2.6 algorithm[4]. Gene quantification was performed using HTSeq[5] and differential expression analysis was performed using edgeR package (glmFit model)[6] in R[7]. Heatmaps with hierarchical tree clustering and

boxplots were created in R with an in-house developed script which is based on ggplot package. Row tree cutting at height of 1.8 was used to obtain discrete clusters of genes with similar pattern of expression across samples. A set of specific gene signatures, which were manually curated from the literature, were retrieved from the RNA sequencing data. A signature was considered significant if >5% of genes had p<0.05. Venn diagrams were created using Venny 2.1.0 online tool[8]. Human-mouse overlap was tested using an online tool based on normal approximation to the exact hypergeometric probability[9].

Enrichment analysis

Significant differentially expressed genes (DEGs) were used for *pathway and gene ontology (GO) analysis* using g:Profiler web-server[10] and ClueGO plug-in in Cytoscape 3.7.0[11 12]. Immunological gene signatures were retrieved from GO-ImmuneSystemProcess-EBI-UniProt-GOA (ClueGO, updated on November 14, 2018). Statistically significant enriched pathways were considered those with Benjamini-Hochberg corrected p-value≤0.05 (two-sided hypergeometric test). *Regulator and transcription factor enrichment* was performed using Regulatory Network Enrichment Analysis[13]. Statistically significant factors were considered those with FC≥1 and p≤ 0.05. *Gene Set Enrichment Analysis (GSEA)*[14] was also performed in order to reveal enriched signatures in our gene sets based on the Molecular Signatures Database (MSigDB) v6.1, and in specific analyses based on publicly available data (see Results section). Gene sets were ranked by taking the –log10 transform of the p-value multiplied by the FC. Significantly upregulated genes were at the top and significantly downregulated genes were at the bottom of the ranked list. GSEA pre-ranked analysis was then performed using the default settings. Enrichment was considered significant by the GSEA software for FDR (q-value) <25%.

Data Sharing Statement

Murine RNA-seq data have been deposited to GEO under accession number GSE128692. Human RNA-seq data have been deposited to EGA database under Study EGAS00001003679; dataset EGAD00001005052.

Grigoriou M, *et al. Ann Rheum Dis* 2020; 79:242–253. doi: 10.1136/annrheumdis-2019-215782

1. Alissafi T, Banos A, Boon L, et al. Tregs restrain dendritic cell autophagy to ameliorate autoimmunity. The Journal of clinical investigation 2017;**127**(7):2789-804 doi: 10.1172/JCI92079[published Online First: Epub Date]|.
2. Isenberg DA, Rahman A, Allen E, et al. BILAG 2004. Development and initial validation of an updated version of the British Isles Lupus Assessment Group's disease activity index for patients with systemic lupus erythematosus. Rheumatology 2005;**44**(7):902-6 doi: 10.1093/rheumatology/keh624[published Online First: Epub Date]|.
3. Andrews S. FastQC A Quality Control tool for High Throughput Sequence Data Secondary FastQC A Quality Control tool for High Throughput Sequence Data http://www.bioinformatics.babraham.ac.uk/projects/fastqc/.
4. Dobin A, Davis CA, Schlesinger F, et al. STAR: ultrafast universal RNA-seq aligner. Bioinformatics 2013;**29**(1):15-21 doi: 10.1093/bioinformatics/bts635[published Online First: Epub Date]|.
5. Anders S, Pyl PT, Huber W. HTSeq—a Python framework to work with high-throughput sequencing data. Bioinformatics 2015;**31**(2):166-69 doi: 10.1093/bioinformatics/btu638[published Online First: Epub Date]|.
6. Robinson MD, McCarthy DJ, Smyth GK. edgeR: a Bioconductor package for differential expression analysis of digital gene expression data. Bioinformatics (Oxford, England) 2010;**26**(1):139-40 doi: 10.1093/bioinformatics/btp616[published Online First: Epub Date]|.
7. R: A language and environment for statistical computing. R Foundation for Statistical Computing. [program], 2018.
8. Oliveros JC. VENNY. An interactive tool for comparing lists with Venn Diagrams. 2007, 2015.
9. Lund J. Statistical significance of the overlap between two groups of genes. Secondary Statistical significance of the overlap between two groups of genes. http://nemates.org/MA/progs/overlap_stats.html.
10. Reimand J, Kull M, Peterson H, et al. g:Profiler--a web-based toolset for functional profiling of gene lists from large-scale experiments. Nucleic acids research 2007;**35**(Web Server issue):W193-200 doi: 10.1093/nar/gkm226[published Online First: Epub Date]|.
11. Bindea G, Mlecnik B, Hackl H, et al. ClueGO: a Cytoscape plug-in to decipher functionally grouped gene ontology and pathway annotation networks. Bioinformatics 2009;**25**(8):1091-3 doi: 10.1093/bioinformatics/btp101[published Online First: Epub Date]|.
12. Shannon P, Markiel A, Ozier O, et al. Cytoscape: a software environment for integrated models of biomolecular interaction networks. Genome research 2003;**13**(11):2498-504 doi: 10.1101/gr.1239303[published Online First: Epub Date]|.
13. Chouvardas P, Kollias G, Nikolaou C. Inferring active regulatory networks from gene expression data using a combination of prior knowledge and enrichment analysis. BMC bioinformatics 2016;**17 Suppl 5**:181 doi: 10.1186/s12859-016-1040-7[published Online First: Epub Date]|.
14. Subramanian A, Tamayo P, Mootha VK, et al. Gene set enrichment analysis: A knowledge-based approach for interpreting genome-wide expression profiles. Proceedings of the National Academy of Sciences 2005;**102**(43):15545-50 doi: 10.1073/pnas.0506580102[published Online First: Epub Date]|.

Grigoriou M, *et al. Ann Rheum Dis* 2020; 79:242–253. doi: 10.1136/annrheumdis-2019-215782
